# Spatial Pattern and Spatial Heterogeneity of Chinese Elite Hospitals: A Country-Level Analysis

**DOI:** 10.3389/fpubh.2021.710810

**Published:** 2021-09-16

**Authors:** Baoguo Shi, Yingteng Fu, Xiaodan Bai, Xiyu Zhang, Ji Zheng, Yuping Wang, Ye Li, Lijun Zhang

**Affiliations:** ^1^Department of Economics, School of Economics, Minzu University of China, Beijing, China; ^2^Research Center of Health Policy and Hospital Management, School of Health Management, Harbin Medical University, Harbin, China; ^3^The Department of Urban Planning and Design, The University of Hong Kong, Hong Kong, China

**Keywords:** elite hospital, spatial pattern, spatial heterogeneity, health resources, geographic weighted regression (GWR)

## Abstract

Elite hospitals represent the highest level of Chinese hospitals in medical service and management, medical quality and safety, technical level and efficiency, which are also one of the important indicators reflecting high-quality medical resources in the region, and their spatial allocation is directly related to the fairness of health resource allocation. We explored the allocation pattern of high-quality resources and its influencing factors in the development of China's health system using geographic weighted regression (GWR), Multi-scale Geographically Weighted Regression (MGWR), GWR and MGWR with Spatial Autocorrelation(GWR-SAR and MGWR-SAR), spatial lag model (SLM), and spatial error model (SEM). The results of OLS regression showed that city level, number of medical colleges, urbanization rate, permanent population and GDP per capita were its significant variables. And spatial auto-correlation of elite hospitals in China is of great significance. Further, its spatial agglomeration phenomenon was confirmed through SLM and SEM. Among them, the city level is the most important factor affecting the spatial allocation of elite hospitals in China. Its action intensity shows a solid and weak mosaic trend in the Middle East, relatively concentrated in some areas with medium intensity and concentrated in the West China. Obviously, China's elite hospitals are unevenly distributed and have evident spatial heterogeneity. Therefore, we suggest that we should pay attention to the spatial governance of high-quality medical resources, attract medical elites in the region, increase investment in medical education in the scarce areas of elite hospitals and develop tele-medicine service.

## Background

Health is the universal desire and basic needs of human beings (1). Everyone has a healthier right (2). Promoting and protecting health is indispensable for human well-being and sustainable economic and social development (3). With the continuous improvement of people's living standards, people's demand for high-quality medical resources becomes more and more urgent. Due to the unequal supply of health service space (4), high-quality medical resources cannot cover the entire population (2), which leads to the lack of access to health service space and directly damages the right to health of some people. The imbalance in the allocation of health resources is not only a worldwide problem, but also one of the major obstacles faced by China's health services (5). At present, China implements a vertical classification system of medical institutions. The standard of hospital classification is the evaluation index of hospital qualification according to hospital function, facilities and technical force. The whole country is unified, regardless of hospital background, ownership, etc. According to the hospital grading management standard, the hospital is determined to be three classes after review, and each class is divided into level A, B and C. In this study, we focus on the top medical institution in China, namely Elite hospital (Class III C-level hospital), as the top medical institution in China, which has great strategic significance for medical and health allocation. At present, China is pursuing the reform of the medical system (6–9). It is of great significance for achieving health equity and protecting citizens' right to health to taking China as an example to study the spatial allocation of elite hospitals, which can also provide a reference for other countries.

Meanwhile, most researches on the spatial allocation of medical resources are implemented by means of geographic information technology. This has become one of the frontier hotpots in international health research (10–13). To our knowledge, the research on the space problem of medical and health resources are relatively rare and mainly focuses on the following three aspects: the fairness of space allocation, space accessibility and hospital location and evaluation. A longitudinal time series study conducted in Tehran (1966–2011) measured the inequality of hospital bed distribution and found that inequality may persist over time and hinder policy initiatives and major political changes (14). Gu also used the ArcGIS network analyst extension module to evaluate the accessibility and fairness of urban medical resources by using medical and health related spatial data (15). A descriptive analysis conducted a hospital Accessibility Study Based on network analysis model, average center and standard distance in ArcGIS environment. It was found that the hospitals were concentrated in the central and southern areas of Kermansha (16). In addition, Shoman evaluated the performance of Istanbul urban investment with the spatial accessibility index to quantify the difficulty of reaching the destination (17). A study in Istanbul, Turkey, used a set method to measure the impact of various factors on the location of health facilities (18). Kim developed an evidence-based decision support system and enhanced it through geographic information system (GIS). The system may overcome the shortcomings of robustness and trend ability (19). The specific research methods mainly include: applying the network analysis model, average center and standard distance in the geographic information system (GIS), combined with remote sensing technology or the 2SFCA (two-step floating catchment area) method extended on the basis of GIS (11, 20) and other methods.

The method above focuses on describing China's spatial pattern of medical resources, measurement of fairness and spatial accessibility, etc., while ignoring the focus on two types of issues: (1) The above method lacked an explanation of causality from a spatial perspective. The traditional regression method uses ordinary least squares (OLS) for model estimation, which requires the data to meet the assumptions of normality, homogeneity of variance, and independence. However, due to the geographical difference of each study area, the study area lacks spatial homogeneity, and thus cannot satisfy the assumption of homogeneity of variance. At the same time, because the regions are not independent, but open to each other, there must be a flow of factors, which cannot satisfy the assumption of independence. Therefore, the existence of spatial effects leads to deviations in OLS estimates. The spatial econometric model can be compensated through the establishment of statistical and econometric relationships between geographic location and spatial connection, providing a new research perspective and analytical work for revealing regional differences and influencing factors (12, 21–23); (2) The above method ignored spatial heterogeneity. Spatial heterogeneity is one of the important properties of spatial data (24, 25), referring to the non-stationary nature of spatial random processes. Ignoring spatial heterogeneity may cause many problems, such as loss of estimation efficiency, biased estimation, and saliency of errors. Geographically weighted regression is an extension of the ordinary linear regression model, which embeds the spatial location of data into the regression equation. By establishing the local regression equation at each point in the spatial range, we can explore the spatial changes and related driving factors of the research object at a certain scale and can be used to predict future results. Because it takes the local effects of spatial objects into account, its advantage is higher accuracy. Based on the above considerations, on the basis of using the Exploratory Spatial Data Analysis (ESDA) method to study the spatial pattern of Chinese elite hospitals, this paper focuses on the analysis of their influencing factors and spatial heterogeneity from the scale of prefectural administrative units. The former uses a spatial regression model, and the latter uses a geographically weighted regression model, with a view to exploring the problems from a spatial perspective, such as the imbalance of high-quality resource allocation in the development of China's medical and health system and its influencing factors, so as to provide reference of the high-quality medical resources allocation for China and other countries.

## Methods

### Research Methods

#### Exploratory Spatial Data Analysis (ESDA)

Our research used spatial autocorrelation to explore the spatial pattern of elite hospitals. Spatial autocorrelation refers to the statistical correlation between a certain attribute values where the distribution of geographical things is different from the spatial position. Generally, the closer the distance is, the greater the correlation between the two values. Generally, Moran's I and Local Moran Index are introduced to measure the global and local spatial correlation features. The former is a method for global clustering test, which tests that the adjacent areas in the entire study area are similar and different (spatial positive correlation, negative correlation), or independent of each other; the latter is used to test whether there are similar or different observations gathered in local areas. Global spatial autocorrelation generally uses Moran's I index (26). Moran's I index is between−1 and 1, and its calculation formula is as follows:


(1) $$\begin{array}{l} I=\frac{n\sum_{i}\sum_{j}W_{ij}\left({\text{X}}_{i}-\bar{\text{X}}\right)\left({\text{X}}_{j}-\bar{\text{X}}\right)}{\left(\sum_{i}\sum_{j}W_{ij}\right)\sum_{i}{\left({\text{X}}_{i}-\bar{\text{X}}\right)}^{2}} \end{array}$$

In the formula: n is the total number of areas in the study; X_*i*_ and X_*j*_ are the numbers of elite hospitals in areas i and j; *W*_*ij*_ are the spatial weight matrix, spatial adjacent is 1 and non-adjacent is 0; $\bar{\text{X}}$ is the average value of numbers of elite hospitals. Perform statistical tests on Moran's I results, usually using *Z*-test:


(2) $$\begin{array}{l} Z\left(I\right)=\frac{\text{I}-\text{E(I)}}{\sqrt{\mathrm{var}\text{(I)}}} \end{array}$$

E(I) is the mathematical expectation, var(I) is the variance.

Local spatial autocorrelation refers to the Local Moran Index (27) of a region to measure the degree of association between Area I and its neighbors. The LISA in this study, as a local measure of spatial autocorrelation (28), was used to identify clusters (i.e., hot or cold spots) and outliers (e.g., regions of neighborhoods with above or below the expected number of elite hospitals). Note that the accumulation of j in the formula does not include the Area I itself, that is, j≠i. A positive I_i_ (I_i_ > 0) means a high value is surrounded by a high value (high-high), or a low value is surrounded by a low value (low-low), which means this element is a cluster; a negative I_i_ (I_i_ < 0) means a low value is surrounded by a high value (low-high), or a high value is surrounded by a low value (high-low), which means this element is an outlier. In any instance, to be regarded as clusters and outliers with statistical significance, the *p*-value of the element must be small enough. The calculation formula is as follows:


(3) $$\begin{array}{l} I_{i}=\frac{\left(\text{X}i-\bar{\text{X}}\right)}{S_{x}^{2}}\sum_{j}\left[W_{ij}\left({\text{X}}_{j}-\bar{\text{X}}\right)\right] \end{array}$$

(4) $$\begin{array}{l} S_{x}^{2}=\sum_{j}\left(X_{j}-\bar{X}\right)/n \end{array}$$

In the formula: n is the total number of areas in the study. X_*i*_ and X_*j*_ are the numbers of elite hospitals in areas i and j; *W*_*ij*_ are spatial weights, $\bar{\text{X}}$ are the average values of the numbers of elite hospitals, $S_{x}^{2}$ are the variances.

#### Spatial Econometric Method

This study mainly uses spatial regression models: Spatial Lag Model (SLM) and Spatial Error Model (SEM)

(1) Spatial Lag Model (SLM) (29)

If the variable concerned has spatial correlation expressed by spatial matrix, only considering its explanatory variable x is not enough to estimate and predict the changing trend of the variable reasonably. Therefore, the spatial lag model assumes the impact caused by the appropriate spatial structure and can better control the effect caused by this spatial effect.


(5) $$\begin{array}{l} Y=\alpha +\rho WY+\beta X+\varepsilon \end{array}$$

where W is the spatial weight matrix of the area; α is a constant term; β are the regression coefficients, which reflects the influence of the explanatory variables change on the explained variable; ρ is the spatial autoregressive coefficient, which is used to measure the spatial spillover effect of the explanatory variable in the geographical vicinity; X is the explanatory variable; ε is the random disturbance term, which is independent and identically distributed.

(2) Spatial error model (SEM) (29). Spatial error model describes spatial perturbation correlation and spatial population correlation. The formula is as follows:

(6) $$\begin{array}{l} Y=\alpha +\beta X+\mu ;\mu =\lambda W\mu +\varepsilon \end{array}$$

where μ is the spatial autocorrelation error term, λ is the autoregressive coefficient of the spatial error term, which measures the degree of influence of the error term of the sample observation value on the explained variable.

(3) Geographically Weighted Regression (30). This method is based on the local regression analysis method and incorporates the spatial location of the data into the regression parameters, and uses the local weighted least square method to estimate point-by-point parameters. The estimated parameters of each spatial unit change with geographic spatial location, thereby directly displaying the spatial heterogeneity of the research object in the research area. Geographically weighted regression can also be regarded as an extension of the traditional global regression model. Its formula is:

(7) $$\begin{array}{l} {\text{y}}_{i}=\beta_{0}\left(u_{i},v_{i}\right)+\underset{k}{\sum }\beta_{k}\left(u_{i},v_{i}\right)x_{ik}+\varepsilon_{i} \end{array}$$

(4) Multi-scale Geographically Weighted Regression (MGWR) (31). The estimated bandwidth (the number of prefecture-level units used for local estimation) of each relationship in GWR is the same, which has some limitations. The recently developed multi-scale geographically weighted regression (MGWR) improves the GWR, which relaxes the assumption of “same spatial scale” and optimizes covariate specific bandwidth. Its formula is:

(8) $$\begin{array}{l} \begin{array}{c} {\text{y}}_{i}=\beta_{\text{h}0}\left(u_{i},v_{i}\right)+\underset{k}{\sum }\beta_{\text{h}k}\left(u_{i},v_{i}\right)x_{ik}+\varepsilon_{i} \end{array} \end{array}$$

(5) GWR-SAR and MGWR-SAR (32). We create a separate GWR Model and MGWR model by adding the spatial lag term in **Equation (5)** to the covariate and combining the model's multi-scale GWR term with the lag dependent variable (MGWR-SAR). We used mgwr2.2 software for all calibrations (https://sgsup.ASU.edu/SPARC/mgwr).

### Data Sources

The article takes China's municipalities directly under the Central Government, sub-provincial cities and prefectures as the research objects (Excluding the data of Hong Kong Special Administrative Region and Taiwan Region). The data of China's elite hospitals were obtained from the Chinese Hospital Level Query System. As of December 31, 2017, China has included 706 elite hospitals. By downloading the latitude and longitude of the elite hospitals, using ARCGIS10.2 and other software, the geographic coordinates of the elite hospital are matched with research regions above. The factors influencing the layout of elite hospitals include economic factors, social factors, political factors, historical factors, and physical geographical factors. The indicators that characterize the level of economic development include GDP per capita and urbanization rate, which comes from the “2017 Statistical Bulletin of Economic and Social Development” of each prefecture-level unit. The indicators that characterize social factors mainly include population size and density, the data of which coming from the “2017 Statistical Bulletin of Economic and Social Development” of each prefecture-level unit. The indicators of historical factors mainly include medical education, that is, the number of medical universities or medical colleges, the data of which coming from the directory of China Medical University (Medical College) and the affiliated hospitals. The political indicator is the city level, that is, the administrative level of the city. The data is obtained from the list of municipalities directly under the Central Government, provincial capital cities, and cities under separate planning. Please refer to Appendix Table 1 for the definition and source of all the above variables.

## Results

### Exploratory Spatial Data Analysis of Elite Hospitals in China

#### Spatial Allocation of Elite Hospitals in China

As of December 31, 2017, a total of 705 elite hospitals had been included in the Chinese hospital ranking query system. From a regional perspective, 283 elite hospitals were located in the eastern coastal area, 166 were located in six central provinces, 171 were located in 12 provinces in the west, and 85 were located in the three provinces of Northeast China. At the provincial level, there were 10 provinces and autonomous regions in China with 30 or more elite hospitals, namely Guangdong (66), Jiangsu (38), Sichuan (36), Hubei (35), Liaoning (34), Jiangxi (33), Hebei (32), Shanxi (32), Heilongjiang (31), Beijing (30); four provinces had five or less elite hospitals: Hainan (5), Yunnan (5), Ningxia (3), Tibet (1). From the city level, there were 16 cities with 10 or more elite hospitals, including 4 municipalities directly under the Central Government, Beijing, Shanghai, Tianjin, and Chongqing, and 12 provincial capital cities. Among them, Beijing, Shanghai and Guangzhou had the largest number of elite hospitals, 30, 24, and 20 respectively, followed by Tianjin (17), Wuhan (17), Xi'an (16), Hangzhou (13), and Taiyuan (13), Nanchang (13), Nanjing (12), Chongqing (11), Chengdu (11), Guiyang (11), Changchun (11), Harbin (10), Fuzhou (10); those with 5–10 elite hospitals There are 17 cities in total, namely Xining (9), Shenyang (9), Foshan (9), Shijiazhuang (8), Nanning (8), Jinan (7), Dongguan (7), Dalian (7), Shenzhen (6), Changsha (6), Lanzhou (6), Hohhot (6), Urumqi (6), Zhengzhou (5), Wuxi (5), Xiamen (5), Jilin (5). Overall, among China's 361 prefecture-level cities (excluding Hong Kong Special Administrative Region, Macau Special Administrative Region and Taiwan), there are 232 cities with one or more elite hospitals. The remaining 129 cities currently do not have elite hospitals. Cities are mostly distributed in Tibet, Ningxia, Yunnan, Hainan, Xinjiang, Qinghai, Gansu, Inner Mongolia, and other provinces.

**Figure 1:**
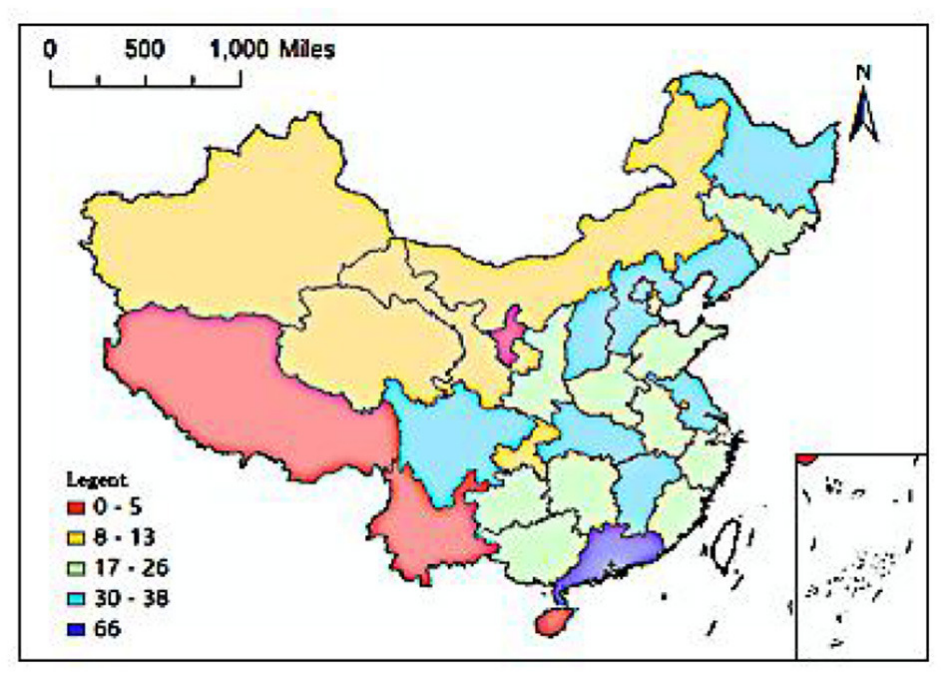
Spatial distribution of provincial unit scale.

**Figure 2:**
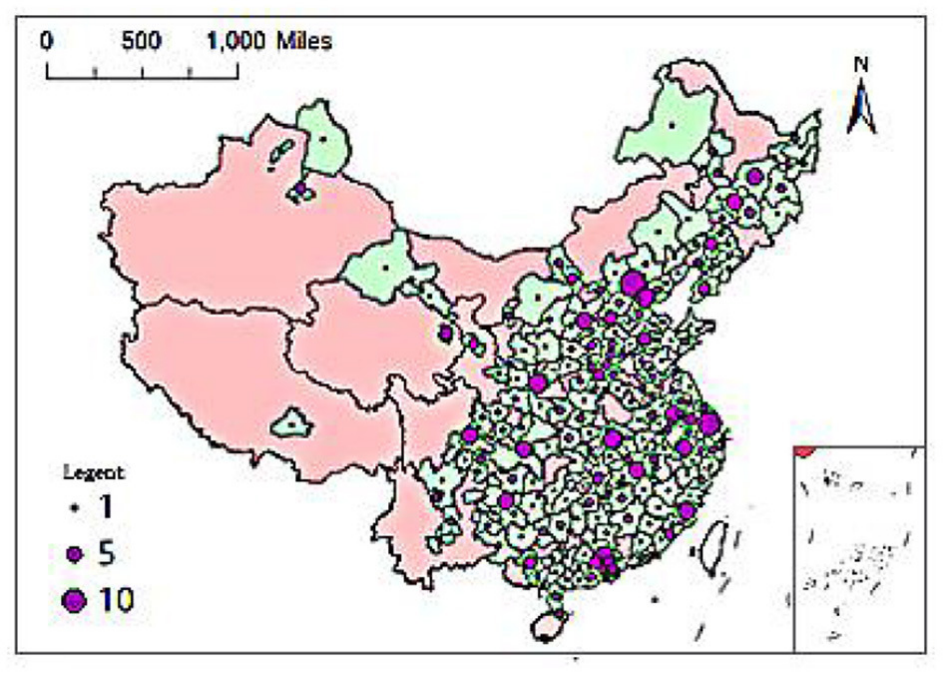
Spatial distribution of city-level unit scale.

Figures 1, 2 show the provincial and municipal spatial allocation patterns of elite hospitals in China. The dotted symbols in the figure indicate the number of elite hospitals. It can be seen from the figure that the number of elite hospitals in China has obvious spatial agglomeration. Elite hospitals are mostly concentrated in the central and eastern regions and northeast regions, while the western region has fewer elite hospitals.

To accurately understand the influencing factors and spatial effects of the allocation of elite hospitals, four spatial weight matrices, including Queen's case and Rook's case, distance threshold weight and *K* nearest neighbors, are used to define the spatial relationship. By comparing the Moran's I statistics calculated by them, the most suitable weight matrix is selected. See Table 1 for specific results.

**Table 1:** Moran's I statistical results of the allocation of elite hospitals in China.

**Weights permutations = 9,999**	**Contiguity weight** **(order** **=** **1)**		**Distance weight**	
	**Queen**	**Rook**	**Threshold (550,000)**	***K*-nearest (4)**
Moran's I	0.0874	0.0874	0.0309	0.0925
E (I)	−0.0028	−0.0028	−0.0028	−0.0028
Mean	−0.0029	−0.0033	−0.0028	−0.0031
Sd	0.0315	0.0318	0.0139	0.0336
*z*-value	2.8690	2.8490	2.4281	2.8410
Pseudo *p*-value	0.0091	0.0093	0.0202	0.0093

It can be seen from the above analysis results that the Moran's I values of elite hospitals calculated by the four methods are all positive, and the calculation results of the contiguity weight matrix are relatively robust. In the correlation test, the *p*-value of Queen is the smallest, and its significance is the strongest. In addition, the queen contiguity matrix often has the advantage of a closer correlation structure with the surrounding areas. Therefore, it is selected to define the spatial relationship.

**Figure 3:**
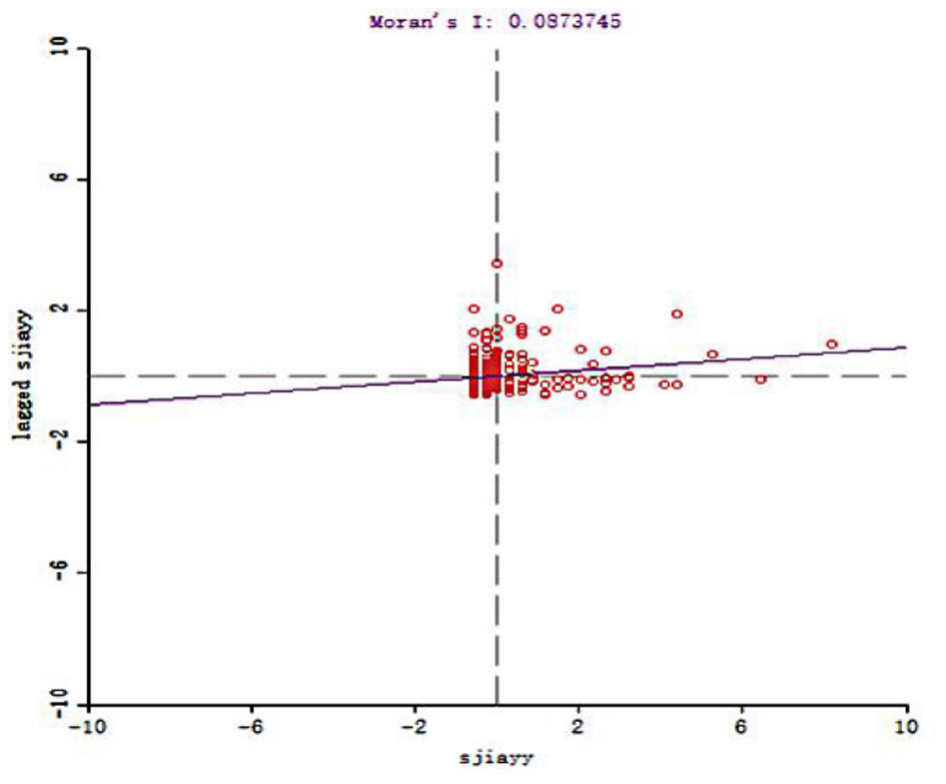
Moran scatter plot of China Elite Hospital.

**Figure 4:**
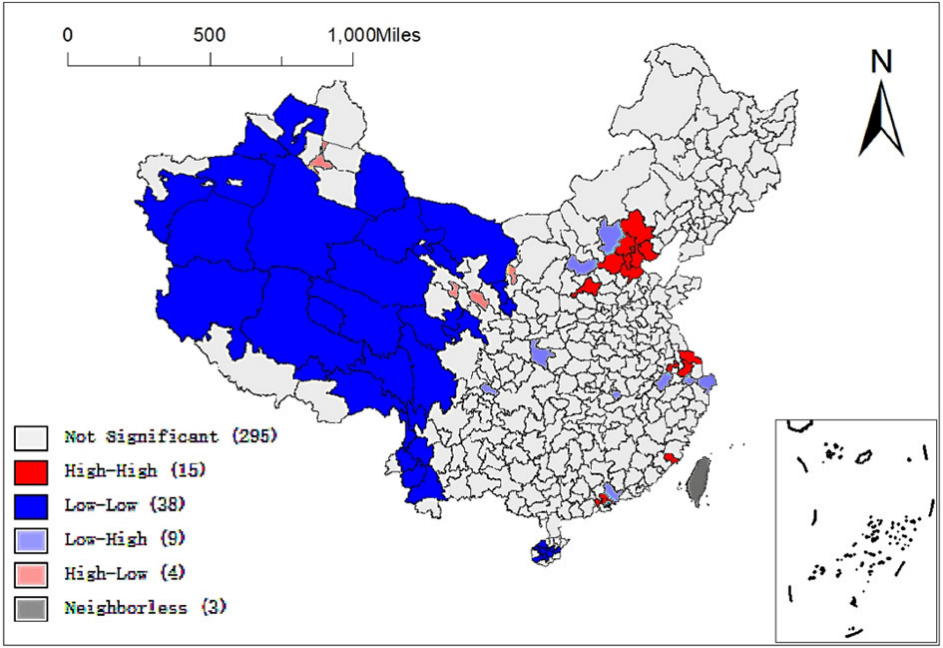
LISA map of China Elite Hospital.

To further clarify the spatial allocation of Chinese elite hospitals, Moran's I statistic was used to measure the spatial autocorrelation of Chinese elite hospitals. The value of the global Moran'I statistic was 0.087 (Figure 3), which indicates that the allocation of elite hospitals across the country was not random, but a definite positive spatial autocorrelation. That is to say, the allocation of elite hospitals in China was clustered and cities with more elite hospitals were usually close geographically and vice versa. Figure 4 and Table 2 described the local spatial autocorrelation clustering phenomenon of elite hospitals in China. The LISA chart demonstrated that the Beijing-Tianjin-Hebei, Yangtze River Delta, and Pearl River Delta regions showed significant high-high aggregation. There was an obvious low-low autocorrelation in the northwest region, which makes an important contribution to the overall positive autocorrelation characteristics of the allocation of elite hospitals in China. However, most cities in the central-eastern and northeastern regions were not significant, and a small number of provincial capital cities showed high-low clusters. In general, the spatial autocorrelation of elite hospitals in China was obvious.

**Table 2:** Cluster statistics of elite hospitals.

**Type**	**Count**	**Prefecture-level city unit**
High-High	15	Beijing, Tianjin, Jinzhong, Chengde, Tangshan, Langfang, Cangzhou, Baoding, Nantong, Changzhou, Suzhou, Putian, Zhongshan, Dongguan, Shenzhen
Low-Low	38	Tacheng area, Yili Kazakh Autonomous Prefecture, Aksu area, Kashgar area, Hotan area, Bayingoleng Mongolian Autonomous Prefecture, Hami area, Ali area, Nagqu area, Lhasa City, Nyingchi area, Qamdo area, Yushu Tibetan Autonomous Prefecture, Haixi Mongolian and Tibetan Autonomous Prefecture, Jiuquan City, Alxa League, Zhongwei City, Guyuan City, Zhangye City, Baoshan City, Nujiang Lisu Autonomous Prefecture, Diqing Tibetan Autonomous Prefecture, Dali Bai Autonomous Prefecture, Lincang City, Pu'er City, Changjiang Li Autonomous County, Baisha Li Autonomous County, Danzhou City, Qionghai City, Qiongzhong Li and Miao Autonomous County, Tunchang County, Baoting Li and Miao Autonomous County, Ledong Li Autonomous County, Wuzhishan City, Guoluo Tibetan Autonomous Prefecture, Huangnan Tibetan Autonomous Prefecture, Gannan Tibetan Autonomous Prefecture, Ganzi Tibetan Autonomous Prefecture
Low-High	9	Zhangjiakou City, Xinzhou City, Ankang City, Ziyang City, Huangshi City, Xuancheng City, Huizhou City, Jiaxing City, Zhoushan City
High-Low	4	Urumqi City, Xining City, Lanzhou City, Yinchuan City

#### Analysis of Spatial Determinants of China Elite Hospital

The OLS model was used for global regression analysis to discuss the spatial allocation of elite hospitals in China and its influencing factors. Based on reading relevant literature and practical experience, as well as the availability of data, the per capita GDP, urbanization rate, permanant population and population density of each region in 2017 are selected to reflect the regional economic and social situation, and the indicators of city level, number of medical colleges and altitude are selected to reflect the regional political, historical and geographical factors, and these variables are determined as explanatory variables. The city level is a binary virtual variable, with 1 assigned to municipalities directly under the Central Government, provincial capital cities and cities listed explicitly in the state plan, and 0 assigned to other cities.

To avoid the huge deviation of regression results due to the collinearity between explanatory variables, the method expansion factor (VIF) is used to test the collinearity of explanatory variables (the results are shown in Table 3). Among all explanatory variables, the statistical significance coefficients at the 5% significance level are GDP, city level, number of medical colleges and urbanization rate. Among them, all VIF are lower than 7.5, indicating that there is no collinearity relationship between variables.

**Table 3:** Results of variable collinearity test.

**Variable**	**Standard error**	**VIF**
City level	0.4153	2.1952
Number of medical colleges	0.1690	2.6779
Urbanization rate	0.0069	1.9974
Permanent population	0.0003	2.0249
GDP per capita	0.0003	1.9670
Population density	0.0002	1.6674
Altitude	0.0001	1.2622

The regression results show that the five variables, GDP per capita, city level, number of medical colleges, permanent population and urbanization rate, are significant at the level of 5%. At the same time, the regression coefficients of GDP per capita, city level, number of medical colleges, permanent population and urbanization rate are positive. This shows that with the improvement of GDP per capita, the increase of the number of medical colleges and permanent population, the rise of city level and the improvement of urbanization rate, the number of elite hospitals in the region will also increase. In addition, the altitude did not pass the significance test. However, the Moran's I for OLS regression model shows a significant spatial effect in the estimation residual. At this time, if the OLS method is directly used, the parameter estimation is only consistent and unbiased but not effective. Therefore, to analyze the spatial influencing factors of the allocation of elite hospitals in China more accurately, the corresponding spatial econometric models SEM and SLM are constructed based on the OLS model for further analysis (results are shown in Table 4). The results show that the significance of the variables of city level, number of medical colleges, GDP per capita, permanent population and population density at 5% level and altitude at 10% level in the SEM model still exists, but the significance of urbanization decreases compared with OLS model. Moreover, the regression coefficients of city level, the number of medical colleges and urbanization rate decrease. The influence of altitude on elite hospitals is negative, but not significant. There is little difference in other coefficient estimations with OLS model. In the SLM model, the significance of the variables of city level, number of medical colleges, permanant population, urbanization rate and GDP per capita still exists, and the regression coefficient of city level and number of medical schools becomes larger, but the significance of population density has disappeared. Compared with the OLS model, both the SEM model and SLM model increase *R*^2^, reduce AIC value. λ and ρ, measuring the coefficient of the error term and spatial lag effect of the dependent variable, were all positive at the significance level of 5%. Therefore, when analyzing the factors affecting the allocation of elite hospitals, it is correct and necessary to consider the spatial effect based on the OLS model. The SEM and SLM model results confirm that there is indeed spatial agglomeration in the allocation of elite hospitals in China. GDP per capita, city level, number of medical colleges, permanent population and urbanization rate have significant positive impacts on the allocation of elite hospitals. In addition, although the SEM and SLM models have been improved compared with the OLS model, in the Lagrange multiplier of the OLS regression model, LM (lag), robust LM (lag), and LM (error) are significant at 10% confidence level, but the *P*-value of robust LM (error) test is >10% significance level, that is, it is not significant. This further illustrates the necessity of adopting the SLM model, which makes the explanation is more convincing.

**Table 4:** Regression estimation results of three models.

	**OLS**		**SLM**		**SEM**	
	**Estimate**	**Standard error**	**Estimate**	**Standard error**	**Estimate**	**Standard error**
Intercept term	−1.0775^**^	0.3489	−1.1898^**^	0.3424	−1.009^**^	0.3546
City level	3.4198^**^	0.4153	3.5789^**^	0.4088	3.3914^**^	0.3993
Number of medical colleges	2.0067^**^	0.1690	2.0133^**^	0.1650	1.9341^**^	0.1627
Urbanization rate	0.0160^**^	0.0069	0.0136^**^	0.0068	0.0122^*^	0.0070
Permanent population	0.0024^**^	0.0003	0.0023^**^	0.0003	0.0024^**^	0.0003
Population density	0.0003^*^	0.0003	0.0003	0.0002	0.0004^**^	0.0002
GDP per capita	7e-006^**^	0.0002	6.47e-006^**^	3.25e-006	9.31e-006^**^	3.40e-006
Altitude	3e-006	0.0001	5.0548e-005	0.0001	−9.0097e-006	0.0001
λ					0.2374^**^	0.7461
ρ			01331^**^	0.4468		
*R* ^2^	0.8203		0.8253		0.8266	
AIC	1,324.1943		1,314.66		1,312.9	
Moran's I (error)	3.2361^**^					
LM (lag)	9.9004^**^					
Robust LM (lag)	3.1209^*^					
LM (error)	8.7240^**^					
Robust LM (error)	2.0446					
Lagrange Multiplier (SARMA)	11.8449^**^					
Log likelihood			−648.33		−648.45	
LR			9.0251^**^		8.7832^**^	

** *Represents 5% significance level*,

* *Represents 10% significance level; binary queen contiguity weight matrix is used*.

In the SLM model, the five variables, GDP per capita, city level, number of medical colleges, permanent population, and urbanization rate, are significant at the level of 5%. Meanwhile, the regression coefficients of these five variables are positive, consistent with the OLS regression results. On the one hand, with the improvement of GDP per capita, the increase in the number of medical colleges and permanent population, the rise of city level and the improvement of urbanization rate, the number of elite hospitals in the region will also increase. On the other hand, the variable regression coefficients of city level, number of medical colleges and city level in the SLM model become larger. That is, in OLS regression, if the city level increases by 1, the number of elite hospitals increases by 3.4198; the number of medical colleges increases by 1, the number of elite hospitals increases by 2.0067. In SLM model, the city level increases by 1, the number of elite hospitals increases by 3.5789; the number of medical colleges increases by 1, the number of elite hospitals increases by 2.0133, which further illustrates the necessity of adopting SLM model.

#### Analysis of Spatial Heterogeneity of Elite Hospitals in China

The above ESDA analysis shows that the allocation of elite hospitals in China shows a significant spatial dependence. To more comprehensively and thoroughly explain the influencing factors of the spatial distribution of elite hospitals in China, especially the spatial heterogeneity of prefecture-level cities, this paper further uses geographical weighted regression for analysis. Before that, this paper will first test the stationarity. The stability test results are shown in Table 5.

**Table 5:** Geographical variability tests of local coefficients.

**Variable**	***F***	**DOF for *F* test**	**DIFF of criterion**
City level	4.7353	2.937 330.014	−7.8764
Number of medical colleges	4.1662	2.419 330.014	−5.0514
Urbanization rate	1.6604	3.664 330.014	2.2508
Permanent population	6.5407	2.642 330.014	−12.140798
Population density	6.6972	2.014 330.014	−9.6638
GDP per capita	2.4049	3.345 330.014	−0.6333
Altitude	2.4942	4.203 330.014	−1.1906

*DIFF of Criterion is positive, indicating that there is no significant spatial variation in variable coefficients*.

It can be seen from Table 5 that the “diff of criterion” values of all explanatory variables except urbanization rate are <0, indicating that they are local variables, while the “diff of criterion” value of urbanization rate is >0, indicating that urbanization rate is a global variable. We will eliminate the global variable, namely “urbanization rate,” to analyze the spatial heterogeneity of local variables.

To explain the determinants of the spatial allocation of elite hospitals in China more comprehensively, especially in the spatial heterogeneity research at the prefecture-level city scale, geographic weighted regression (GWR) is further used to analyze the influence degree and spatial difference of the seven determinants (city level, number of medical colleges, permanent population, population density, GDP per capita, and urbanization rate) selected in OLS model. When using geographic weighted regression, to achieve the purpose of more optimization, we use GWR, MGWR, GWR-SAR, and MGWR-SAR models, respectively. The regression results are shown in Table 6. The weight we select is the reciprocal of distance, the model type is “Gaussian,” the spatial kernel type is “Adaptive Bisquare,” the bandwidth type is “golden section,” and “AICc” is selected as the optimization criterion. By comparing AICc term and *R*^2^ term, it can be seen that AICc is the smallest and *R*^2^ is the largest in MGWR-SAR model, indicating that MGWR-SAR model is the best choice.

**Table 6:** Regression results of four models.

	**GWR**	**MGWR**	**GWR-SAR**	**MGWR-SAR**
Residual sum of squares	48.926	48.568	47.080	46.947
AICc	394.931	368.625	396.963	361.054
*R* ^2^	0.866	0.867	0.870	0.871
Log likelihood	−151.248	−149.910	−144.351	−143.839

**Figure 5:**
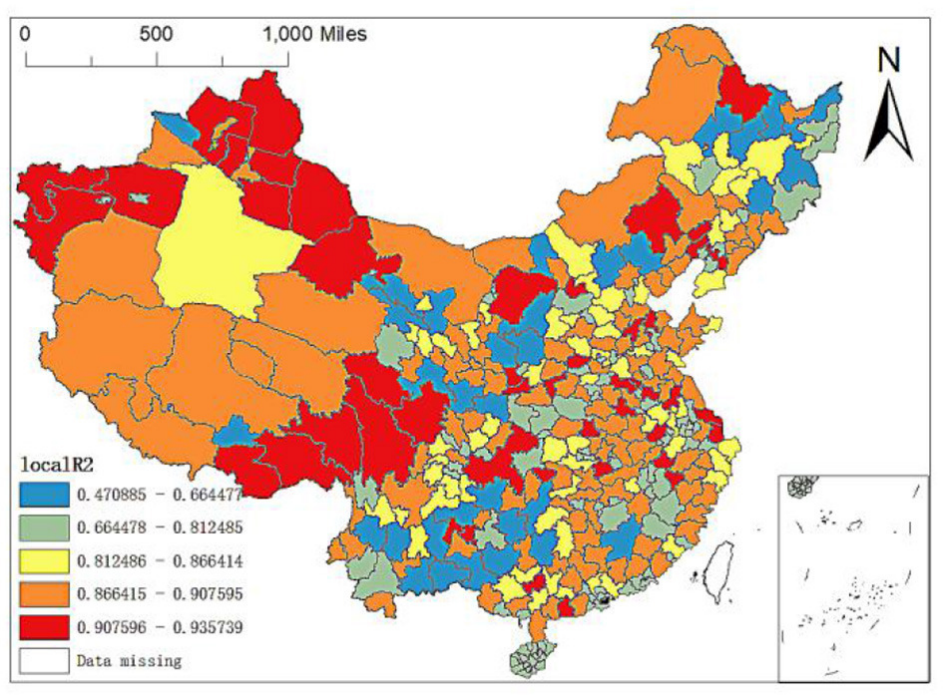
The spatial allocation of the local *R*^2^ of the MGWR-SAR model.

**Figure 6:**
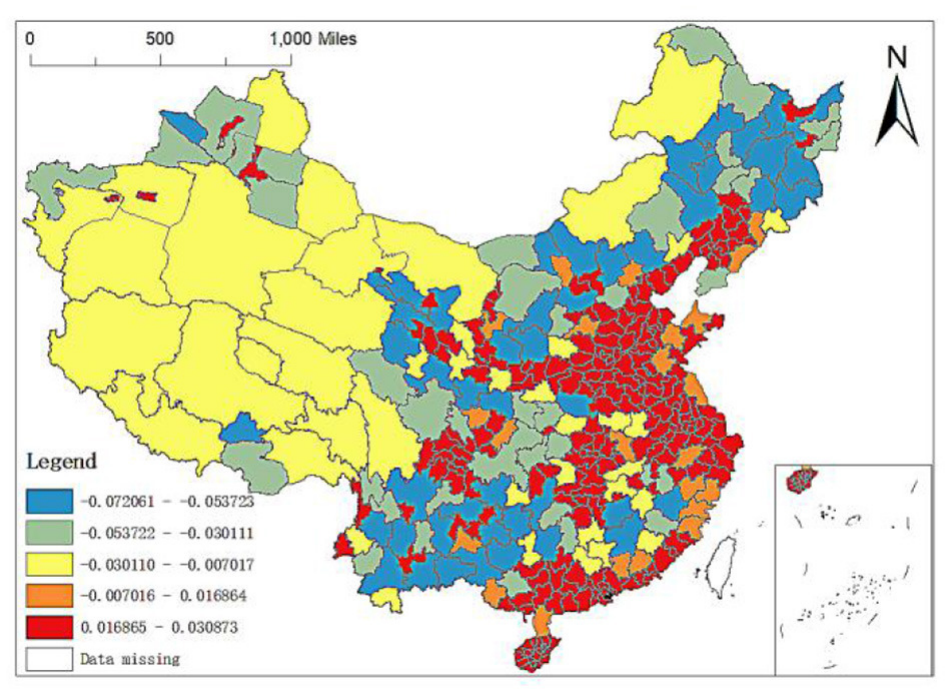
Spatial distribution of regression coefficients of altitude.

**Figure 7:**
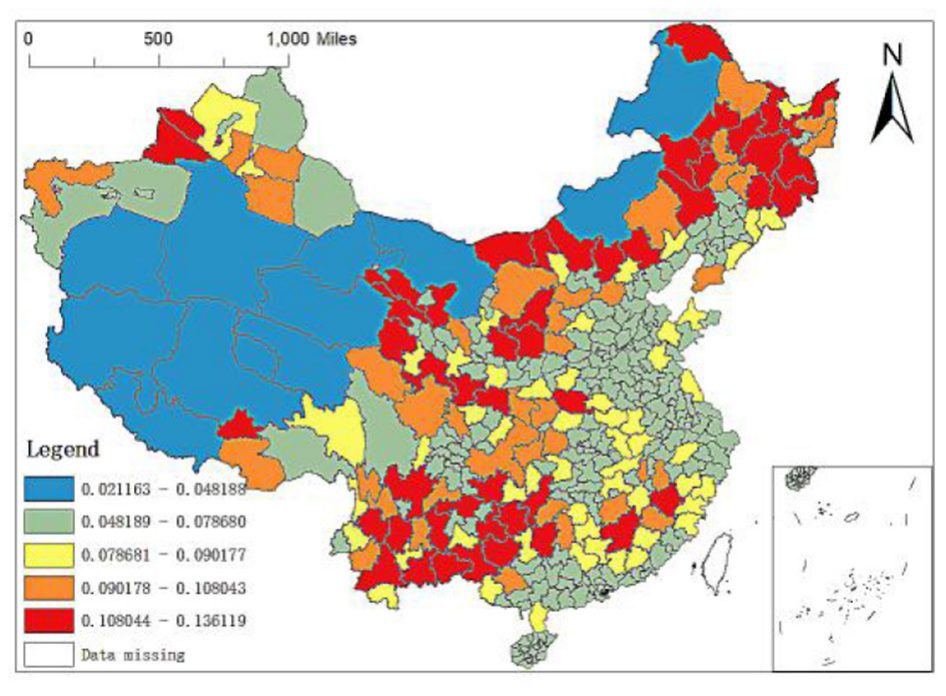
Spatial distribution of regression coefficients of GDP per capita.

**Figure 8:**
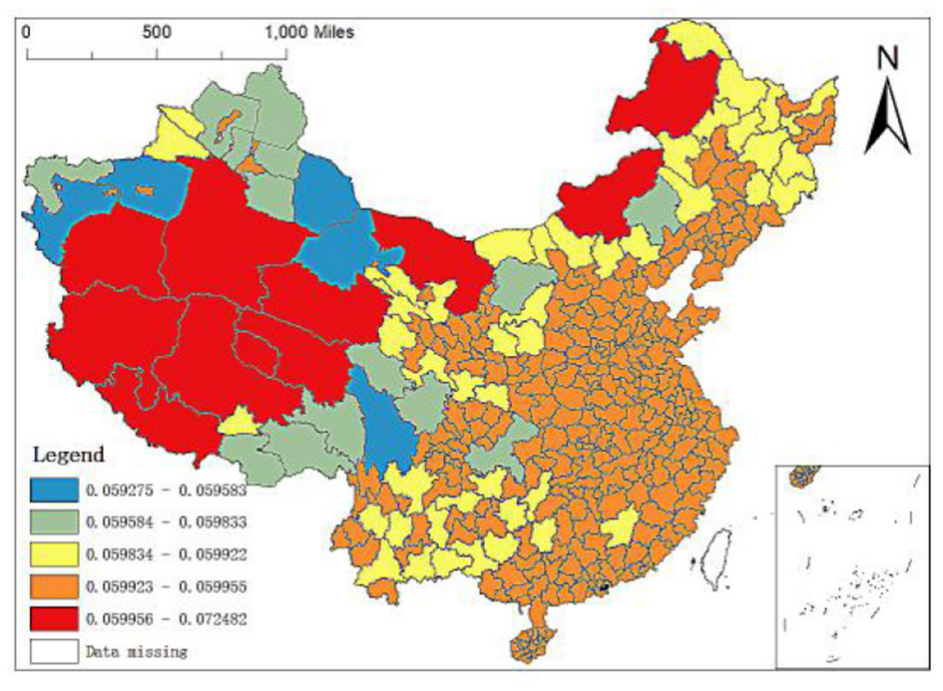
Spatial distribution of regression coefficients of population density.

**Figure 9:**
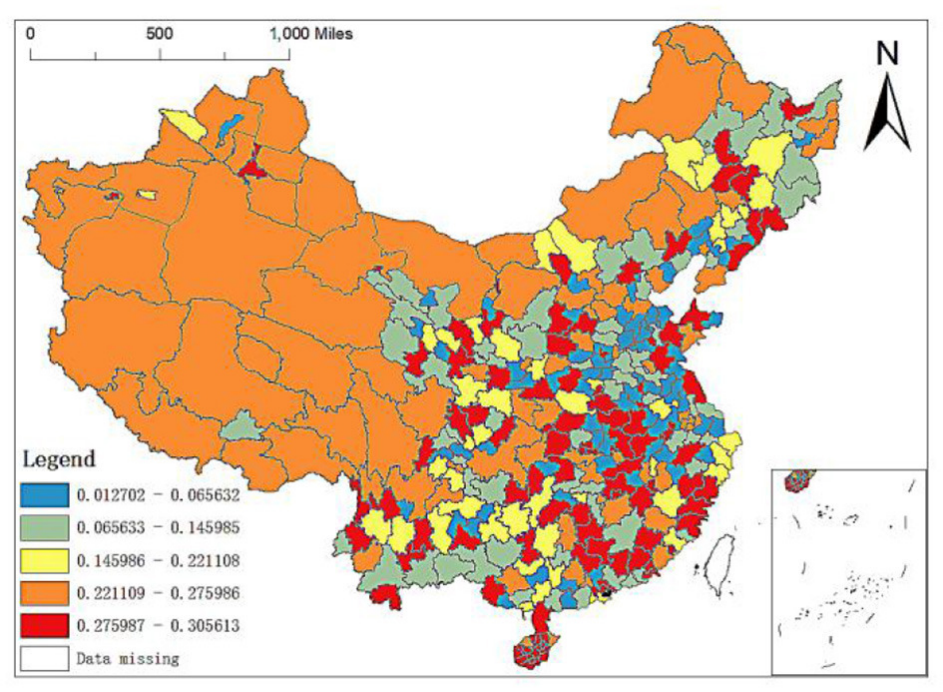
Spatial distribution of regression coefficients of permanent population in MGWR-SAR model.

**Figure 10:**
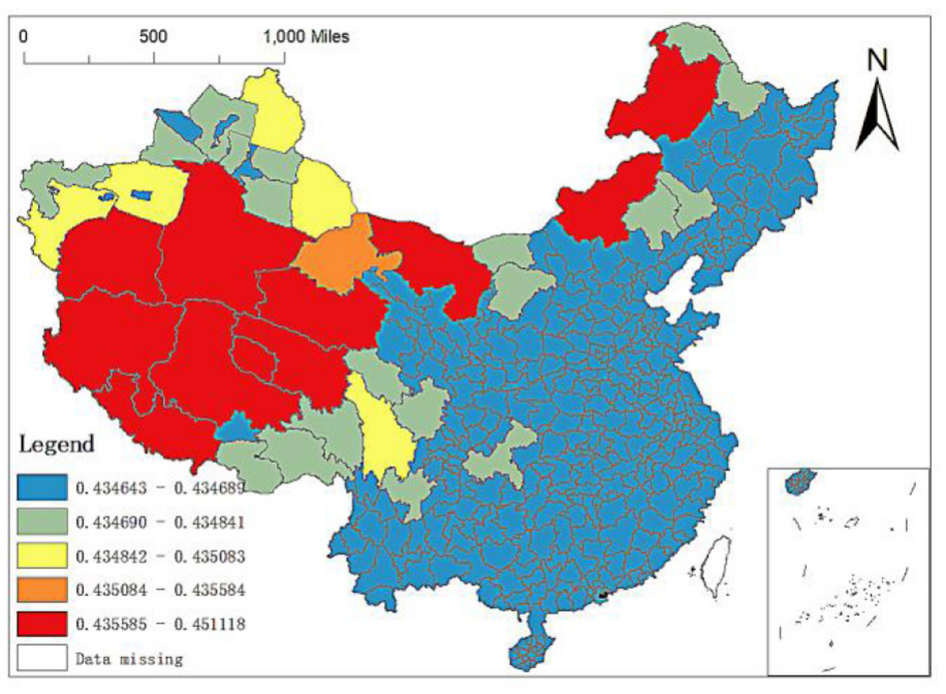
Spatial distribution of regression coefficients of medical colleges in MGWR-SAR model.

In the MGWR-SAR model, the termination criterion of MGWR-SAR is 1.0e-05, and the number of iterations used is 32. Table 7 lists summary statistics of the local parameter estimates generated by MGWR-SAR, and they are displayed in full in Figures 5–10. The second column of Table 7 shows the local variable name; the second shows the bandwidth of each local variable; the third shows the mean (Mean), minimum (Min), and maximum (Max) values of the local parameter estimates of each covariate. The fourth column indicates a classification of coefficients based on *t*-tests, adjusted for multiple hypothesis testing (33), including the proportion of significant coefficients (*p* ≤ 0.05), the proportion of significant positive coefficients to significant coefficients (+), and the proportion of significant negative coefficients to significant coefficients (–).

**Table 7:** Parameter estimates for the regression of number of elite hospitals using MGWR-SAR.

		**MGWR-SAR coefficient**			**Saliency (95%) percentage of cities divided**		
**Variable**	**Bandwidth**	**Mean**	**Min**	**Max**	***P* ≤ 0.05 (%)**	**+ (%)**	**– (%)**
Intercept term	362.000	0.012	0.002	0.012	0.000	100.000	0.000
City level	43.000	0.274	−0.036	0.812	60.606	100.000	0.000
Number of medical colleges	362.000	0.435	0.435	0.451	100.000	100.000	0.000
Permanent population	111.000	0.191	0.013	0.307	69.697	100.000	0.000
Population density	362.000	0.060	0.059	0.072	97.245	100.000	0.000
GDP per capita	318.000	0.083	0.021	0.136	94.766	100.000	0.000
Altitude	298.000	0.003	−0.072	0.031	4.132	100.000	0.000

The results show that the intercept term of all cities is not significant. For two-thirds of cities, the impact of city level and permanent population on elite hospitals is significant, and higher city level or permanent population is related to higher elite hospitals. For more than 90% of cities, population density and GDP per capita impact on elite hospitals is significant, and higher population density or GDP per capita is related to higher elite hospitals. It is worth noting that the impact of the number of medical colleges on elite hospitals is significant for all cities, and the impact of the number of medical colleges on elite hospitals is positive. Finally, the altitude of <5% of cities significantly affects elite hospitals, and this impact is also positive.

To more clearly show the impact of GDP per capita, permanent population, population density, altitude, number of medical schools and city level on the spatial allocation of China's elite hospitals, we will explain it in detail through its spatial allocation characteristic map.

First, the spatial difference of local *R*^2^: the maximum local *R*^2^ is 0.9357, and the minimum value is 0.4709. There are apparent spatial differences in local *R*^2^. As shown from Figure 5, the model's explanatory variables have stronger explanatory power to the north of Xinjiang and partial cities in Southwest China than other regions. In contrast, the explanatory power to Yunnan, Guangxi and some cities in Northeast China is relatively weak, and the explanatory power to Zhangjiakou in Hebei is the weakest. Therefore, according to the size of local *R*^2^, this paper divides China's prefecture level units into five levels: (1) local *R*^2^ is between 0.4709 and 0.6645, mainly distributed in some cities in Yunnan, Guangxi and Guizhou, Zhangye, Wuwei, Qingyang, Longnan, Hanzhong, Yulin, Yan'an and Tongchuan in Northwest China, Qiqihar, Suihua, Mudanjiang, Yichun, Jiamusi and Jilin in Northeast China, Lhasa in Tibet, Bortala Mongolian Autonomous Prefecture in Xinjiang, Baotou in Inner Mongolia, Chengde and Zhangjiajie in Hebei, and Ganzhou in Jiangxi; (2) Local *R*^2^ is between 0.6645 and 0.8125, mainly distributed in Middle East China, such as most cities of Hainan, partial cities in Jiangxi Hubei and Guangdong; in addition, regression values of Lincang, Pu'er and Diqing Tibetan autonomous prefectures in Yunnan and Shuangyashan, Jixi, Yanbian Korean Autonomous Prefecture, Baicheng in Northeast China are all in the interval; (3) Local *R*^2^ is between 0.8125 and 0.8664, mainly distributed in Baoding, Shijiazhuang and Xingtai in Hebei, Ulanqab and Xing'an League in Inner Mongolia, Datong, Luliang, Changzhi and Taiyuan in Shanxi, Bayingolin Mongolian Autonomous Prefecture in Xinjiang, Harbin, Songyuan, Changchun, Tieling, Shenyang, and Dalian in Northeast China, most cities in Sichuan and Zhoushan, Ningbo and Taizhou in Zhejiang; (4) Local *R*^2^ is between 0.8664 and 0.9076, mainly distributed in some cities in Inner Mongolia, Shandong, Qinghai, Ningxia, Henan, Anhui, Hunan, Hubei and Zhejiang, Hotan region in Xinjiang, Ili, Karamay, Urumqi, Shigatse region, Naqu region and Ali Region in Tibet and Hegang, Daxinganling region, Dandong, Benxi, Baishan and Fushun in Northeast China; (5) Local *R*^2^ is between 0.9076 and 0.9357, mainly concentrated in Xinjiang, Ordos and Chifeng in Inner Mongolia, Jiuquan in Gansu. In addition, the regression values of Ganzi Tibetan Autonomous Prefecture and Aba Tibetan and Qiang Autonomous Prefecture in Sichuan, Golog Tibetan Autonomous Prefecture in Qinghai, Shannan region, Nyingchi region and Changdu region in Tibet, Chongqing and Shanghai are also relatively high, belonging to this range. At the same time, the cities with high regression values are also scattered in the Middle East China. In particular, the local *R*^2^ in Huai'an City, Jiangsu Province is the highest.

Secondly, the regression coefficient of altitude: altitude refers to the vertical distance between the ground and the sea level. Usually, the higher the altitude, the thinner the air, and the less suitable for human habitation and life. As can be seen from Figure 6, the maximum value of regression coefficient of altitude is 0.0309 and the minimum value is −0.0721. The prefecture level units with regression coefficient ranging from −0.0721 to 0.0070 are widely distributed (i.e., the distribution of the first three lower coefficient grades in Figure 6), including the whole west, most of the northeast and most of Middle South, while the prefecture level units with regression coefficient ranging from −0.0070 to 0.0309 are mainly distributed in the Middle East and coastal areas. Especially, the regression coefficients of Zhoushan, Ningbo and Wenzhou in Zhejiang, Weihai and Qingdao in Shandong and Zhangzhou, Shanwei and Jieyang in Guangzhou are pretty large. However, in OLS regression, the regression results of altitude are not significant, indicating that they have no great impact on the spatial distribution of elite hospitals in China.

Third, the regression coefficient between GDP per capita, population density and permanent population. It can be seen from the OLS results above that although the GDP per capita and population density have significant impacts on the spatial distribution of elite hospitals in China, the regression coefficient is small. The GDP per capita and population density positively affect the distribution of elite hospitals, and their coefficients are 6.54e-006 and 0.0003, respectively. From Figure 7, it can be found that the GDP per capita level in some cities in Northeast China, some cities in Southwest China, most cities in Gansu, Ganzhou in Jiangxi, Nanping in Fujian, Yulin in Shaanxi, Yan'an, Zhangjiakou and Chengde in Hebei, Wulanchabu, Baotou and Bayannur in Inner Mongolia have large influence coefficients on the distribution of elite hospitals. The impact coefficients of GDP per capita in the western and eastern regions are small, especially in some cities in Xinjiang, Tibet and Inner Mongolia, which is basically in the range of 0.0212–0.0482.

Population density is the number of people per unit land area, which is an essential factor to investigate the economic development of a country or region and an important symbol to measure the environmental pressure of a country or region. Therefore, this paper puts population density into the analysis framework. Figure 8 shows the spatial distribution of the regression coefficient of population density of MGWR-SAR model. It can be found that the regression coefficient of population density is positive, which indicates that the increase of regional population density will have a positive impact on the number distribution of local elite hospitals. In addition, comparing Figures 7, 8, it is found that the spatial distribution of the population density regression coefficients is just opposite to that of the GDP per capita regression coefficients. Specifically, the coefficients of population density in the eastern region are large, but those of GDP per capita are relatively low; while in most areas in West China, the coefficients of population density are large, but the coefficients of GDP per capita are relatively small.

The permanent population refers to the population who often lives at home or have been at home for more than 6 months throughout the year, with the characteristics of relative stability. It can be seen from the OLS results above that the permanent population also has a significant impact on the spatial distribution of elite hospitals in China. Still, the regression coefficient is also relatively small, which positively impacts the distribution of elite hospitals with the coefficient of 0.0024. From the spatial distribution map of the regression coefficient of the resident population of the MGWR-SAR model (Figure 9), it can be seen that the regression coefficients of the western region are between 0.2211 and 0.2760, the grade of its influence coefficients is in the middle and even high position. The strong and weak effects of the regression coefficients of the resident population in the central and southeast regions are inlaid. In addition, the distribution of elite hospitals in China is related to the population size, but the relationship is not close. Population density has little impact on the number distribution of elite hospitals, which also lays a foundation for us to continue to find more influential factors.

To sum up, the distribution of elite hospitals in China has a certain relationship with social and economic development, but the relationship is not close. The GDP level per capita, population density and permanent population at the prefectural-city level in China have little impact on the number distribution of elite hospitals, indicating that other factors will have substantial effects on the number distribution of elite hospitals in China.

Fourth, the regression coefficient of the number of medical colleges: the OLS regression results above show that when other explanatory variables remain unchanged, the number of medical colleges increases by one standard unit and the number of elite hospitals increases by 2.0067 standard units. The number of medical colleges is the core variable to characterize medical education. At present, there are few studies on the unbalanced allocation of high-quality medical resources in China from the perspective of medical education. By the end of 2017, 84 medical universities or medical schools in China were mainly distributed in the eastern region. Among the 361 prefecture-level city units, 19 cities have two or more medical schools, including six medical colleges in Beijing and Shanghai, five or four medical colleges in Guangzhou and Shenyang, and three medical schools in Tianjin, Hangzhou and Changchun. Generally, there is at least one Affiliated Hospital under the medical college. The hospital is often the best local medical institution, which can explain that the number of medical colleges is one of the main factors affecting the allocation of elite hospitals in China. Still, the difference in the number of medical colleges between regions leads to the spatial characteristics of its influence coefficients on the allocation of elite hospitals.

Through the analysis of MGWR-SAR model, it can be found that the minimum impact coefficient in prefecture-level units is 0.4346, and the maximum is 0.4511. It can be seen that there are spatial differences in the impact of the number of medical colleges on the allocation of elite hospitals in different regions, although the spatial differences are pretty small. From the spatial distribution of the regression coefficient (Figure 10), the action intensity shows a trend of being stronger in the West and weaker in the East: (1) The regression coefficients of the whole Middle East region, Tacheng, Turpan, Changji, Urumqi, Ili, Karamay, Bortala Mongolian Autonomous Prefecture, Kizilsu Kirgiz Autonomous Prefecture in Xinjiang and Lhasa, Shannan, Nyingchi and Changdu regions in Tibet are small, especially the cities in the whole Middle East region. This shows that the number of medical schools has little impact on the distribution of elite hospitals in the Middle East, Xinjiang, and Tibet; (2) The regression coefficients of cities under the jurisdiction of Ganzi Tibetan Autonomous Prefecture in Sichuan, Altay region in Xinjiang, Hami region, Aksu region, and Kashgar region are between 0.4351 and 0.4356, indicating that the number of medical colleges has higher impacts on the spatial distribution of elite hospitals in these regions than cities under the jurisdiction of the Middle East. However, they are lower than bayingol Hotan region in Xinjiang, Ali Region and Naqu region in Tibet; (3) The number of medical colleges has the greatest impact on the distribution of elite hospitals in parts of Tibet, southern Xinjiang and Alxa, Xilingol and Hulunbuir in Inner Mongolia, and their regression coefficients are between 0.4356 and 0.4511. The cities with large influence coefficients in Tibet are mainly Ali Region, Naqu region and Xigaze region, and the cities with large influence coefficients in Xinjiang are bayingol Mongolian Autonomous Prefecture and Hotan region. Among them, the maximum value of influence coefficient appears in bayingol Mongolian Autonomous Prefecture in southern Xinjiang.

**Figure 11:**
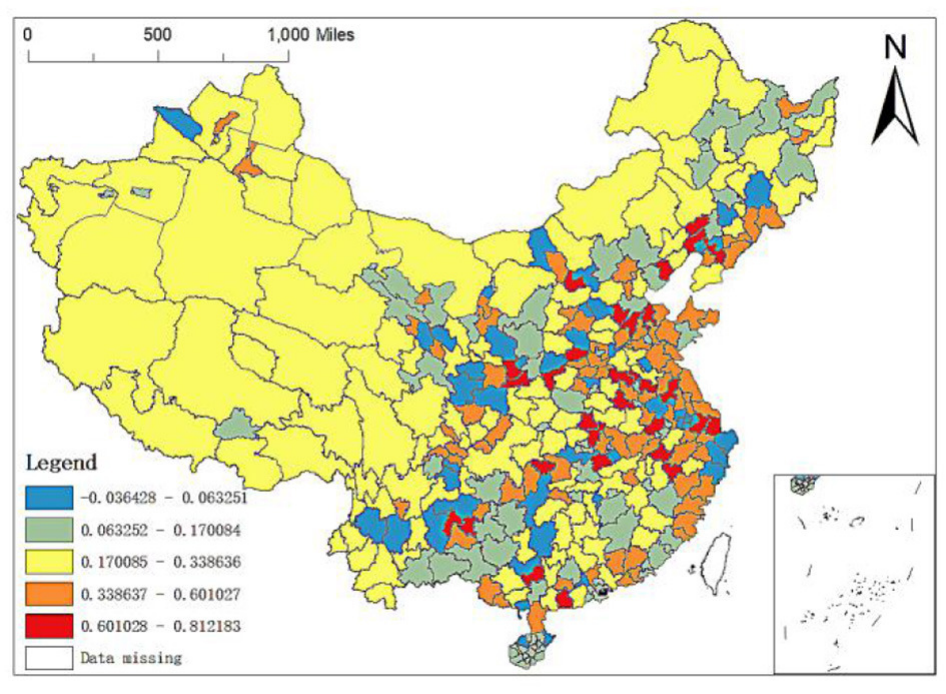
Spatial distribution of regression coefficients of the number of city level in the GWR model.

Finally, city level regression coefficient: there is relatively little literature on the spatial allocation of high-quality medical resources in China from the urban level. In this paper, the research units are divided into two types: prefecture level units and units above prefecture level. Units above the prefecture level include municipalities directly under the central government, provincial capital cities and cities specifically designated in the state plan. According to experience, it can be judged that the higher the city level is, the more elite hospitals there are. The statistical results also confirm this judgment. According to the OLS analysis results, the regression coefficient of city level is 3.4198, which is much higher than other variables. Therefore, the city level is the most crucial factor affecting the spatial allocation of elite hospitals in China. From the spatial distribution of the regression coefficient (Figure 11), its action intensity shows a solid and weak mosaic trend in the Middle East, relatively concentrated in some areas with medium intensity and concentrated in the West China. The specific performance is as follows: the impact coefficients of provincial capital cities or cities with higher urban levels in various provinces in the Middle East region are the highest, such as Shanghai, etc. In addition, Xi'an, as the capital city of Shaanxi Province in Northwest China, has also a high impact coefficient at the city level. The western region's influence coefficient is in the middle position, ranging from 0.1701 to 0.3386, while it is low in Hainan, Bortala Mongolian Autonomous Prefecture in Xinjiang, some cities in Hebei, some areas in Northeast and Southwest China, Zhoushan, Ningbo, and Taizhou in Zhejiang. Therefore, it is necessary to increase the investment in medical resources in non-provincial capital cities, to weaken the impact of city level on the uneven spatial allocation of elite hospitals and then alleviate the uneven spatial allocation of high-quality medical resources.

## Discussion

This article uses 2017 China prefecture-level unit data to describe the inequality of spatial allocation in elite hospitals and explore its determinants. We have drawn the spatial layout map, spatial clustering map and spatial heterogeneity map of China's elite hospitals summarizing three main research findings.

First of all, despite the ongoing medical reform in China, the spatial agglomeration of high-quality medical resources is significant, and there is a serious inequality in its geographical distribution. This study uses ESDA to identify the degree of spatial agglomeration of elite hospitals in China, and uses Moran'I statistics to prove a significant positive spatial autocorrelation, indicating that the allocation of elite hospitals in China shows obvious spatial agglomeration. From a regional perspective, elite hospitals are mostly concentrated in the eastern coastal region (283), the central region (166) and the northeast region (85); The Beijing-Tianjin-Hebei, Yangtze River Delta, and Pearl River Delta regions are the most significant high-high agglomeration areas in the country, while the western region shows significant low-low agglomeration. At the city level, elite hospitals are mainly distributed in the four municipalities directly under the Central Government, Beijing, Tianjin, Shanghai, and Chongqing, and near the capital cities of the central and eastern regions. Elite hospitals in many cities in the western region are relatively scarce. Prefecture-level cities in high-high agglomeration areas not only have more elite hospitals in this city, but also have more in neighboring areas, while prefecture-level cities in low-low agglomeration areas are just the opposite. It shows that the regional imbalance of China's high-quality medical resources is obvious. Some scholars found that China's health resources are mainly distributed in economically developed regions (34), and 46.68% of the elite hospitals are concentrated in the eastern coastal areas (35). Even some studies (36) in China have directly confirmed similar hot spot analysis results. Interestingly, even at the prefectural-city level, the distribution of high-quality medical resources still tends to areas with high economic level (37). This imbalance is largely due to China's long-term supply side resource allocation, which tends to place a large number of high-quality medical resources in economically developed regions. However, underdeveloped areas, including rural areas, are often located in remote areas with high operating costs and poor local economic development, which lead to local residents becoming victims of China's health inequity. At the same time, this explains to a large extent the phenomenon of “difficult medical treatment” in China. Over the past 20 years, the focus of China's medical reform is to establish and improve the medical insurance system. It is hoped that insurance can improve the economic accessibility of health services, while spatial accessibility is neglected. This is very detrimental to patients' equal access to quality medical services. With economic development and family income growth, as well as the high incidence of major diseases, residents are increasingly demanding high-quality medical and health services, hoping to seek medical treatment in elite hospitals. A large number of patients are chasing high-quality medical resources and health services across regions, resulting in the phenomenon of “difficult medical treatment” (38).

Secondly, through the analysis of SLM model, it is found that the factors influencing the allocation of elite hospitals in China include GDP per capita, city level, number of medical colleges, urbanization rate and permanent population. All of the variables have positive impacts on the number of elite hospitals. As previous studies found, the registered residence population has a positive effect on the allocation of medical resources (36), which may be because the expansion of health service demand caused by the increase of population size is one of the factors considered by the government in the allocation of health resources. In addition, a previous study confirmed the correlation between population density and public service facilities, which is also popularized for elite hospitals (39). Similar studies also show that high-level cities with high-quality economic conditions are easy to attract high-quality medical resources (34). Even in the western region, the problem of “capital city concentration” will be more prominent (40). Taking Beijing, Shanghai and Guangzhou (the most developed cities in China) as examples, numbers of elite hospitals in these three cities are the largest. Among them, Beijing is the capital of China (also the economic center of Beijing Tianjin Hebei), Shanghai is the economic center of Yangtze River Delta, and Guangzhou is the economic center of Pearl River Delta. The reasons for this phenomenon may be as follows: (1) the financial capacity to implement the goal of health resource allocation is different among regions, which makes the allocation of high-quality health resources different among different levels of cities or cities with different economic conditions. (2) At the same time, it is very possible for medical colleges and universities to be the important influencing factors of elite hospitals in China. From the above examples, Beijing, Shanghai and Guangzhou are also cities with the highest level of medical education in China, with the largest number of medical universities or colleges in China. Due to the unique training mode of Chinese doctors, the medical college usually has at least one affiliated hospital, and this hospital is often the best local medical institution. As of the end of 2017, there were 84 medical universities or medical colleges in China, mainly in the eastern region. On the one hand, these affiliated hospitals absorb graduates from medical colleges and universities, on the other hand, they serve as practice places to cultivate talents for medical colleges and universities. This circulation mode makes it easier for good medical colleges and universities to obtain high-quality affiliated hospitals, and thus creates a centralized allocation of high-quality medical resources.

Third, the MGWR-SAR model confirms that the factors affecting the allocation of elite hospitals in China are spatially heterogeneous. Among them, GDP per capita and population density can promote the number of regional elite hospitals, but the spatial distribution of regression coefficient is just the opposite. In some areas such as some cities in East and most cities in West China, the regression coefficient of population density is relatively high, while the regression coefficient of GDP per capita is relatively small. The influence coefficients of the number of medical colleges on the allocation of regional elite hospitals are between 0.4346 and 0.4511. Its spatial difference is small and its effect degree shows a trend of strong in the West and weak in the East. The city level has the greatest influence on the allocation of elite hospitals, and for different prefecture-level city units, the degree of influence has obvious spatial changes. For example, Hainan and some cities in Northeast and Southwest are less affected, while Xi'an and other provincial capitals, Shanghai and other high-level cities have greater influence. The existence of spatial heterogeneity indicates that there is no “one size fits all” in regulating the spatial layout of China's high-quality medical resources, and targeted intervention measures should be implemented for different cities.

Our research has its limitations. The first limitation is that the number of elite hospitals in each city is adopted as the core indicator to measure high-quality medical resources. Although it is an intuitive indicator, it does not consider factors such as the scale of elite hospitals and service quality. In the empirical process, it is found that the GDP per capita and urbanization rate have an impact on elite hospitals, but the regression coefficient is small. Therefore, only using the number of elite hospitals as the core variable of high-quality medical resources may underestimate the impact of factors such as GDP per capita and urbanization rate. The second limitation is that it does not take into account the impact of floating patients or patients seeking medical care across regions (9). Beijing, Shanghai, and Guangzhou are the cities with the highest concentration of elite hospitals in China with the most migrant patients. The high-quality medical resources in these cities are often squeezed by “outsiders.” Take Beijing as an example, the annual number of migrant patients in Beijing is about 220 million, and more than 600,000 out-of-town patients come to Beijing for treatment every day. However, the large number of migrant patients or patients seeking medical care across regions further proves the imbalance in the spatial allocation of elite hospitals in China. People have to move across regions to obtain high-quality medical facilities/treatment. Finally, we noticed that the analysis in this article is horizontal, only using the cross-sectional data of China's prefecture-level units in 2017, so it is impossible to demonstrate the changing trends of economic and social factors affecting elite hospitals. In summary, our research confirms the reality of high spatial agglomeration of elite hospitals in China, indicating that China's high-quality medical resources have unequal spatial allocation, especially the extreme shortage of elite hospitals in the western region, which threatens the realization of health equity. The fairness of the spatial allocation of high-quality medical resources should be ensured, and the rights of every citizen to enjoy high-quality medical resources should be protected.

## Conclusion

The current imbalance in the spatial allocation of high-quality medical resources in China has become a severe constraint to coordinate regional development and social equity and justice. Based on the above analysis, we make the following recommendations. First of all, attention should be paid to the spatial governance of high-quality medical resources. At the national level, it is necessary to formulate long-term plans for the construction of elite hospitals in underdeveloped cities, adjust the spatial layout of high-quality medical resources, and recommend at least one elite hospital in a prefecture-level city to alleviate the spatial imbalance in the allocation of high-quality medical resources between regions. Secondly, attracting outstanding medical talents to regions where elite hospitals are scarce. A series of incentives such as high salaries, job title evaluation, education and training can be used to attract outstanding medical professionals to “sink” to regions where elite hospitals are scarce. Third, increasing investment in medical education in areas where elite hospitals are scarce. Relying on universities and scientific research institutions in regions where elite hospitals are scarce, it is necessary to increase investment in medical education, improve the availability of local high-quality medical resources and establish the affiliated hospitals. Fourth, we should establish a benign competition elimination mechanism for elite hospitals, improve their exit criteria, and realize the dynamic planning of health resources based on multiple influencing factors. Finally, developing the telemedicine services. With the rapid development of 5G, AI, blockchain, big data and other technologies, it provides technical support for the establishment of a partial or even a whole system of telemedicine networks. In the future, with the remoteization of core medical services and the systematization of telemedicine, the issue of spatial accessibility of high-quality medical services will be effectively resolved.

## Data Availability Statement

The original contributions presented in the study are included in the article/Supplementary Material, further inquiries can be directed to the corresponding author/s.

## Author Contributions

BS: writing—original draft, data analysis, and framework design. YW, JZ, and XZ: data collection and literature retrieval. XB, XZ, and YF: revision. YL and LZ: writing—review and editing. All authors contributed to the article and approved the submitted version.

## Funding

This research was funded by Humanities and Social Sciences Foundation of Ministry of Education of China (Grant No. 19YJCGAT004), National Social Science Foundation of China (Grant No. 20BGJ026), and National Natural Science Foundation (71874045, 71403073, and 72174047).

## Conflict of Interest

The authors declare that the research was conducted in the absence of any commercial or financial relationships that could be construed as a potential conflict of interest.

## Publisher's Note

All claims expressed in this article are solely those of the authors and do not necessarily represent those of their affiliated organizations, or those of the publisher, the editors and the reviewers. Any product that may be evaluated in this article, or claim that may be made by its manufacturer, is not guaranteed or endorsed by the publisher.

## Abbreviations

ESDA: Exploratory Spatial Data Analysis
GIS: Geographic Information System
GWR: Geographic Weighted Regression
OLS: Ordinary least Squares
SEM: Spatial Error Model
SLM: Spatial Lag Model
2SFCA method: Two-Step Floating Catchment Area method.
