# Supplementary material for: Spatial Pattern and Spatial Heterogeneity of Chinese Elite Hospitals: A Country-Level Analysis
Source: Front Public Health. 2021 Sep 16;9:710810. doi: 10.3389/fpubh.2021.710810 (PMC8481595; doi:10.3389/fpubh.2021.710810)
Supplement: Supplementary file 1 [file Table_1.DOCX]

Appendix Table 1. Table of variable definition and source

| **Variable** | | **Definition** | **Unit** | **Data source** |
| --- | --- | --- | --- | --- |
| Independent Variables | Altitude | The vertical distance of a point on the ground above sea level | Mile | Government websites of administrative units |
|  | Permanant population | Residents or floating population living in their city who have lived at home for more than 6 months in a year | Ten thousand people | 2017 Statistical Bulletin of Economic and Social Development announced by the government of each administrative unit |
|  | Urbanization rate | Urban population/ Permanant population | % | 2017 Statistical Bulletin of Economic and Social Development announced by the government of each administrative unit |
|  | GDP per capital | Gross Domestic Product per capital | Yuan per capital | 2017 Statistical Bulletin of Economic and Social Development announced by the government of each administrative unit |
|  | Number of medical colleges | Number of medical colleges in an administrative area | N/A | List of Chinese universities published by the Ministry of Education |
|  | Population density | Population per unit land area | Person per square kilometre | 2017 Statistical Bulletin of Economic and Social Development announced by the government of each administrative unit |
|  | City level | All cities are divided into two criteria: value=1：Prefecture-level area value=2：City specifically designated in the state plan or municipality directly under the Central Government | N/A | Chinese city level standards |
| Dependent Variable | Number of elite hospitals | Number of elite hospitals in an administrative area | N/A | Chinese Hospital Level Query System |
